# Identification of 5-HT receptor subtypes enhancing inhibitory transmission in the rat spinal dorsal horn *in vitro*

**DOI:** 10.1186/1744-8069-8-58

**Published:** 2012-08-20

**Authors:** Du-Jie Xie, Daisuke Uta, Peng-Yu Feng, Masahito Wakita, Min-Chul Shin, Hidemasa Furue, Megumu Yoshimura

**Affiliations:** 1 Graduate School of Health Sciences, Kumamoto Health Science University, Kumamoto, 861-5598, Japan; 2 Department of Integrative Physiology, Graduate School of Medical Sciences, Kyushu University, Fukuoka, 812-8582, Japan; 3 Department of Information Physiology, National Institute for Physiological Sciences, Okazaki, 444-8787, Japan

**Keywords:** IPSC, 5-HT receptor, Substantia gelatinosa, Presynaptic release

## Abstract

**Background:**

5-hydroxytryptamine (5-HT) is one of the major neurotransmitters widely distributed in the CNS. Several 5-HT receptor subtypes have been identified in the spinal dorsal horn which act on both pre- and postsynaptic sites of excitatory and inhibitory neurons. However, the receptor subtypes and sites of actions as well as underlying mechanism are not clarified rigorously. Several electrophysiological studies have been performed to investigate the effects of 5-HT on excitatory transmission in substantia gelatinosa (SG) of the spinal cord. In the present study, to understand the effects of 5-HT on the inhibitory synaptic transmission and to identify receptor subtypes, the blind whole cell recordings were performed from SG neurons of rat spinal cord slices.

**Results:**

Bath applied 5-HT (50 μM) increased the frequency but not amplitudes of spontaneous inhibitory postsynaptic currents (sIPSCs) in 58% of neurons, and both amplitude and frequency in 23% of neurons. The frequencies of GABAergic and glycinergic mIPSCs were both enhanced. TTX (0.5 μM) had no effect on the increasing frequency, while the enhancement of amplitude of IPSCs was eliminated. Evoked-IPSCs (eIPSCs) induced by focal stimulation near the recording neurons in the presence of CNQX and APV were enhanced in amplitude by 5-HT. In the presence of Ba^2+^ (1 mM), a potassium channel blocker, 5-HT had no effect on both frequency and amplitude. A 5-HT_2A_ receptor agonist, TCB-2 mimicked the 5-HT effect, and ketanserin, an antagonist of 5-HT_2A_ receptor, inhibited the effect of 5-HT partially and TCB-2 almost completely. A 5-HT_2C_ receptor agonist WAY 161503 mimicked the 5-HT effect and this effect was blocked by a 5-HT_2C_ receptor antagonist, N-desmethylclozapine. The amplitudes of sIPSCs were unaffected by 5-HT_2A_ or 5-HT_2C_ agonists. A 5-HT_3_ receptor agonist mCPBG enhanced both amplitude and frequency of sIPSCs. This effect was blocked by a 5-HT_3_ receptor antagonist ICS-205,930. The perfusion of 5-HT_2B_ receptor agonist had no effect on sIPSCs.

**Conclusions:**

Our results demonstrated that 5-HT modulated the inhibitory transmission in SG by the activation of 5-HT_2A_ and 5-HT_2C_ receptors subtypes located predominantly at inhibitory interneuron terminals, and 5-HT_3_ receptors located at inhibitory interneuron terminals and soma-dendrites, consequently enhanced both frequency and amplitude of IPSCs.

## Background

The descending inhibitory system composed mainly of the periaqueductal gray and consecutive reticular formation is a structure modulating the nociceptive transmission from periphery to the central nervous system (CNS). 5-HT is one of the main neurotransmitters of the descending system [1,2] which terminates preferentially on superficial laminae (Laminae I and II), especially the substantial gelatinosa (SG, lamina II). The SG is composed of interneurons and plays as a local circuit for processing nociceptive transmission. The 5-HT system originates from the rostral ventromedial medulla (RVM) including the nucleus raphe magnus, projects to the spinal cord through the dorsolateral funiculus and modulates the nociceptive transmission by interacting with 5-HT receptor subtypes. Exact mechanisms and receptor subtypes modulating nociceptive transmission are, however, still obscure [3-6].

The receptor of 5-HT has been classified into seven distinct classes (5-HT_1_-5-HT_7_), some of these are further divided into subtypes, through pharmacological and molecular biological studies [7-9]. The 5-HT receptors are G protein coupled, with exception of the 5-HT_3_ receptor which is a ligand gated ion channel [10-12]. Some of the subtypes are found in the spinal cord, existing at presynaptic or postsynaptic loci of excitatory or inhibitory SG neurons [13-17]. The autoradiographic studies show that 5-HT_1A_, 5-HT_1B_, 5-HT_1D_, 5-HT_2A_, 5-HT_2C_, 5-HT_3_ and 5-HT_7_ receptors are distributed in the superficial laminae of the spinal cord [6,18-23]. RT-PCR study shows that in DRG all subtypes of 5-HT receptors could be detected except for 5-HT_1E_, 5-HT_2B_ and 5-HT_5B_[24]. Although there is controversy regarding the contribution of 5-HT receptor subtypes on the sensory transmission, there are at least four families of 5-HT receptors (5-HT_1_, 5-HT_2_, 5-HT_3_ and 5-HT_7_) have been shown to modulate the nociceptive transmission [25,26]. Behavioral examinations show that stimulation of RVM or intrathecal administration of agonists of 5-HT_2_ or 5-HT_3_ receptor mediates antinociception on such as formalin test [27-30]; paw pressure test [31,32] and hot plate tests [33]. These effects are blocked by intrathecal administration of 5-HT_2_ or 5-HT_3_ receptor antagonist. There are also reports showing an pronociceptive responses of 5-HT [11,13,34].

*γ* - aminobutyric acid (GABA) and glycine are major inhibitory neurotransmitters in the spinal cord [35-37]. Inhibitory synaptic transmission mediated by GABA and glycine plays an important role in the modulation and integration of nociceptive sensory transmission [38-40]. GABA-like and glycine-like immunoreactive neurons exist in the spinal dorsal horn, with fibers and terminals densely distributed in the SG. GABA and glycine coexisting neurons are also observed in the SG [41-45].

5-HT activates different subtypes of receptors on the inhibitory neurons in the spinal dorsal horn, resulting in the modulation of the nociceptive transmission. Previous electrophysiological studies [13,14,46] show possible mechanisms underlying the 5-HT effects in the superficial dorsal horn. First, 5-HT directly activates postsynaptic 5-HT_1A_ receptor and induces an outward current, inhibiting excitatory neurons and subsequently producing the analgesic effect [13]. Second, 5-HT induces an inward current in the small population of SG neurons through the activation of postsynaptic 5-HT_3_ receptors on inhibitory interneurons [13,47]. Third, 5-HT inhibits glutamate release from C afferent fibers by activating presynaptic 5-HT_1A_-like receptors and shows an inhibitory effect on nociception [14]. In this study, not only inhibitory but also excitatory effects on glutamatergic transmission are reported, 5-HT transiently inhibits a frequency of mEPSCs and then enhances. Fourth, 5-HT acts on inhibitory interneurons and enhances the release of GABA and/or glycine. The receptor subtypes and sites of actions as well as underlying mechanism are, however, not clarified rigorously. In the present study, using the blind whole cell recording technique, the effects of 5-HT on the synaptic transmission were studied in SG to identify the receptor subtypes responsible for the enhancement of the inhibitory transmitter release.

## Results

### Effects of 5-HT on sIPSCs and mIPSCs in the spinal substantia gelatinosa

**Figure 1:**
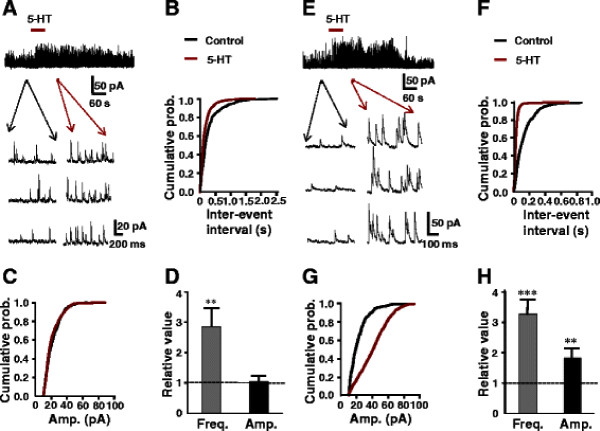
**Effects of 5-HT on sIPSCs recorded at the holding potential of 0 mV.** (**A**) Bath applied 5-HT (50 μM) increased a frequency but not amplitude of sIPSCs in 58% of SG neurons. Lower traces show sIPSCs before (left) and after (right) the application of 5-HT in the expanded time scale. (**B**) cumulative sIPSCs of the inter-event interval (**P < 0.01, K-S test) and amplitude (**C**, P > 0.05, K-S test) distributions recorded in control and in the presence of 5-HT. (**D**) shows the relative frequency and amplitude compared with the pre-application levels (n = 98). (**E**) 5-HT (50 μM) increased the frequency and amplitudes of sIPSCs in 23% of SG neurons. Lower typical traces of sIPSCs observed before, during application of 5-HT. (**F**) Cumulative probability of the inter-event interval and amplitude (**G**) of sIPSCs for the neuron in (E). Both distributions of amplitude and frequency during 5-HT were significantly different from the control (***P < 0.001 and **P < 0.01, respectively, K-S test). (**H**) The bar graph normalized results of the frequency and amplitude with the control (n = 36).

The membrane potential was hold at 0 mV to observe the effects of 5-HT on sIPSCs in SG. Perfusion of 5-HT (50 μM) for 60 s resulted in two different effects in the total of 168 neurons tested. In 58% (98/168) neurons, significant increase in a frequency of sIPSCs from 4.4 ± 1.8 Hz to 12.9 ± 2.6 Hz (paired *t*-test, *P* < 0.01) by 5-HT was observed without a change in a amplitude of 12.6 ± 1.1 to 13.1 ± 1.2 pA (*P* > 0.05, Figure 1A). The same results were depicted in inter-event intervals and cumulative histograms of the sIPSCs amplitudes (Figure 1B, C). The averages of the relative frequency and amplitude were 285% and 104% of the control, respectively (Figure 1D). While in 23% (36/168) of neurons, 5-HT significantly increased both frequency and amplitude of sIPSCs from 5.2 ± 1.0 Hz and 11.2 ± 1.3 pA, respectively, to 16.7 ± 2.3 Hz (*P* < 0.001) and 20.4 ± 1.8 pA (n = 36, *P* < 0.01 Figure 1E) and also on the cumulative distributions (Figure 1F, G). The averages of the relative frequency and amplitude were 324% and 182% of the control, respectively, (Figure 1H).

**Figure 2:**
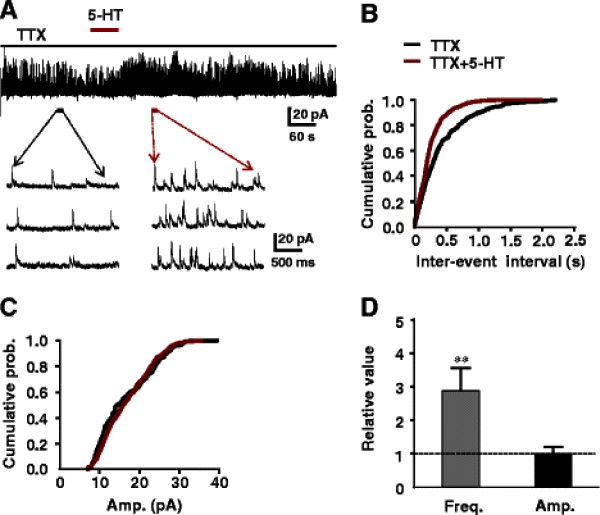
**Effects of 5-HT on mIPSCs.** (**A**) Effect of 5-HT (50 μM) on mIPSCs in the presence of TTX (0.5 μM). Frequency, but not amplitude of mIPSCs increased following 5-HT application. Lower typical traces of mIPSCs were taken before (control) and after the onset of 5-HT application in the presence of TTX. (**B, C**) Cumulative probability of the inter-event interval and amplitude of mIPSCs were obtained from the neuron (A). (**D**) The change in frequency during 5-HT was significantly different from control (**P < 0.01, K-S test), the amplitude during 5-HT was not different from the control (K-S test, P > 0.05, n = 5).

Next, the effects of 5-HT on mIPSCs were examined. In the presence of 0.5 μM TTX, the effect of 50 μM 5-HT simply increased the frequency but not the amplitude of mIPSCs (Figure 2A). mIPSCs changed from 1.8 ± 0.3 Hz in frequency and 14.2 ± 2.1 pA in amplitude to 5.2 ± 0.7 Hz (*P* < 0.01) and 14.7 ± 2.2 pA, respectively (*P* > 0.05, n = 5). The same results were depicted in inter-event intervals of the mIPSCs and cumulative histograms of the mIPSCs amplitudes, (Figure 2B, C). The averages of the frequency and amplitude of mIPSCs to the pre-application levels of 5-HT were 289% and 104%, respectively (Figure 2D).

**Figure 3:**
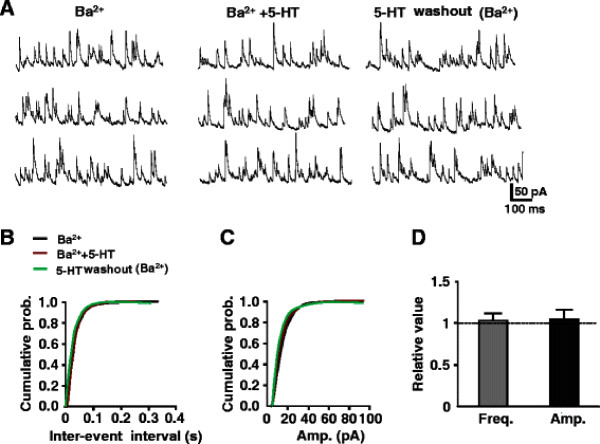
**Effects of 5-HT on mIPSCs in the presence of Ba**^**2+**^. (**A**) Typical traces of mIPSCs observed before (control), during, and after the application of 5-HT (50 μM) in the presence of Ba^2+^ (1 mM). (**B**) Cumulative mIPSCs of the inter-event interval and (**C**) amplitude distributions compiled from the trace indicated in (A). Both the frequency and amplitude during 5-HT was not different from the control. (**D**) The bar graph shows the relative of the mIPSCs frequency and amplitude (paired *t*-test, P > 0.05, n = 7).

The effects of 5-HT on mIPSCs of SG neurons were further studied in the presence of TTX (0.5 μM) and Ba^2+^ (1 mM), a potassium channel blocker. The frequency of mIPSCs in SG neurons was increased markedly by superfusion of slices with 1 mM BaCl_2_. 50 μM 5-HT did not affect the frequency of mIPSCs further more in this condition. The IPSCs were from 21.9 ± 3.6 Hz and 22.5 ± 3.7 pA to 21.7 ± 4.2 Hz (*P* > 0.05) and 22.4 ± 4.0 pA *(P* > 0.05, n = 7, Figure 3A), respectively. The same results were depicted in inter-event intervals and cumulative histograms of the mIPSCs amplitudes (Figure 3B, C). In the presence of Ba^2+^, the averages of frequency and amplitude to the pre-application levels of 5-HT were 103% and 104%, respectively (Figure 3D). These results suggested that the effects of 5-HT on inhibitory transmission were concerned with potassium channels; 5-HT blocked the potassium channels at the inhibitory interneuron terminals and in some cases soma-dendrites and subsequently enhance the release of inhibitory neurotransmitters.

### Effects of 5-HT on GABAergic and Glycinergic mIPSCs

Spontaneously occurring mIPSCs were recorded from SG neurons in the presence of TTX (0.5 μM) and CNQX (10 μM). GABAergic and glycinergic interneurons are distributed extensively in the superficial dorsal horn. The effects of 5-HT on the mIPSCs mediated by GABA or glycine were studied.

**Figure 4:**
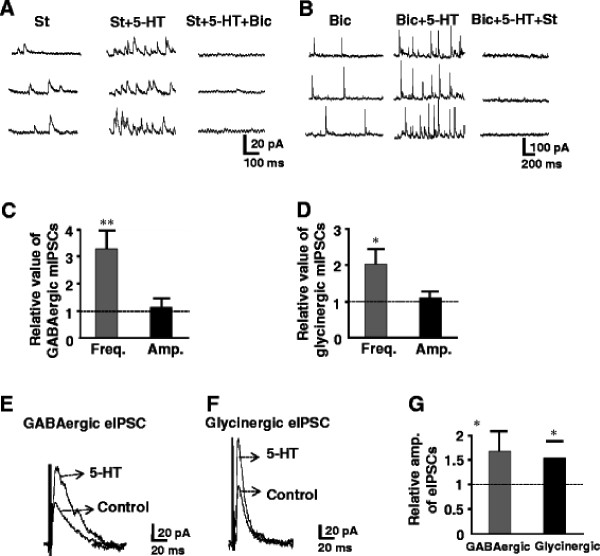
**Effects of 5-HT on mIPSCs and eIPSCs of GABAergic and glycinergic interneuron.** (**A**) 5-HT (50 μM) increased the frequency of GABAergic mIPSCs in the presence of strychnine (St, 2 μM). Subsequent application of bicuculline (Bic, 10 μM) completely eliminated the remaining mIPSCs. (**B**) 5-HT (50 μM) also increased the frequency of glycinergic mIPSCs in the presence of bicuculline (10 μM). The facilitated mIPSCs were eliminated by the application of strychnine (2 μM). The bar graph shows the relative frequency and amplitude for GABAergic (**C**, n = 7) and glycinergic (**D**, n = 5) mIPSCs, respectively. (paired *t*-test, ** P < 0.01 and * P < 0.05). (**E**) 5-HT (50 μM) increased the amplitude of GABAergic (n = 8) eIPSCs with the control in the presence of strychnine (2 μM) together with CNQX (10 μM) and APV (50 μM). (**F**) 5-HT (50 μM) also increased the amplitude of glycinergic (n = 7) eIPSCs with the control in the presence bicuculline (10 μM) together with CNQX and APV. (**G**) The bar graph shows the relative amplitude for GABAergic and glycinergic eIPSCs (paired *t*-test, * P < 0.05 and * P < 0.05).

In the presence of 2 μM strychnine, 5-HT (50 μM) significantly increased the remaining mIPSCs frequency from 5.2 ± 1.7 Hz to 16.9 ± 2.1 Hz (n = 7, *P* < 0.01), without affecting of current amplitude from 13.4 ± 2.5 pA to 15.3 ± 2.7 pA (n = 7, *P* > 0.05). These mIPSCs were blocked by additional administrations of 10 μM bicuculline, confirming that facilitated mIPSCs were GABAergic (Figure 4A). The averages of the relative frequency and amplitude were 328% and 114% of the control, respectively (Figure 4C). Likewise, in the presence of 10 μM bicuculline, 5-HT also increased the remaining mIPSCs in frequency from 3.8 ± 1.1 Hz to 7.7 ± 1.3 Hz (*P* < 0.05), without affecting of current amplitude from 29.3 ± 4.4 pA to 32.2 ± 4.7 pA (n = 5, *P* > 0.05). This effect was blocked by simultaneous perfusion of 2 μM strychnine and 10 μM bicuculline (Figure 4B). The averages of the relative frequency and amplitude were 203% and 110% of the control, respectively (Figure 4D). Thus, the results demonstrated that 5-HT increased the frequencies of both GABAergic and glycinegic mIPSCs. Next, the effects of 5-HT on evoked GABAergic and glycinergic eIPSCs were studied in the presence of CNQX (10 μM) and APV (50 μM). The results showed that the amplitudes of both GABAergic (n = 8) and glycinergic (n = 7) eIPSCs were enhanced. The amplitudes of eIPSCs were from 54.4 ± 12.4 pA and 91.4 ± 7.4 pA to 91.4 ± 14.5 pA (*P* < 0.05) and 140.8 ± 9.8 pA (*P* < 0.05), respectively (Figure 4E, F). The averages of the relative amplitude were 168% and 154% of the control, respectively (Figure 4G).

### Identification of 5-HT receptor subtypes in enhancement of the inhibitory transmitter release

**Figure 5:**
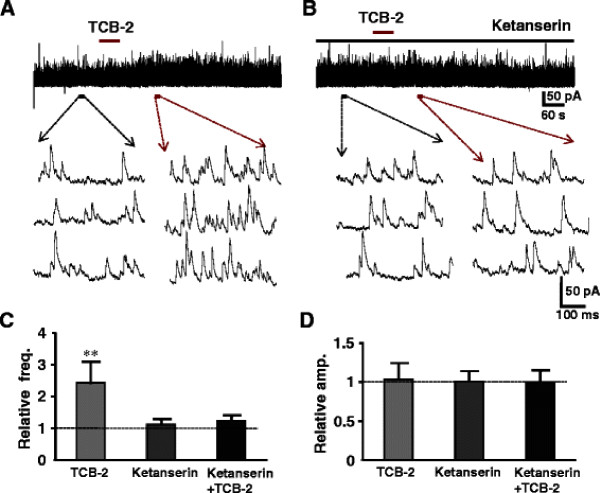
**Effects of 5-HT2A receptor agonist and antagonist on sIPSCs.** (**A**) Representative recording showing the effects of 5-HT2A receptor agonist TCB-2 (10 μM) mimicked the enhancing effects of 5-HT (50 μM) on sIPSCs, increasing the frequency, but not amplitude. Lower traces show sIPSCs before (left) and after (right) the application of TCB-2 in the expanded time scale. (**B**) Shows 5-HT2A receptor antagonist ketanserin (10 μM) on the effects of TCB-2 on sIPSCs recorded from the same neuron (A). Lower traces shows sIPSCs taken before control (ketanserin) and the after TCB-2 application in the presence of ketanserin. The bar graph shows the relative frequency (**C**) and amplitude (**D**) for TCB-2, ketanserin and TCB-2 + ketanserin, respectively (paired *t*-test, ** P < 0.01, n = 7).

**Figure 6:**
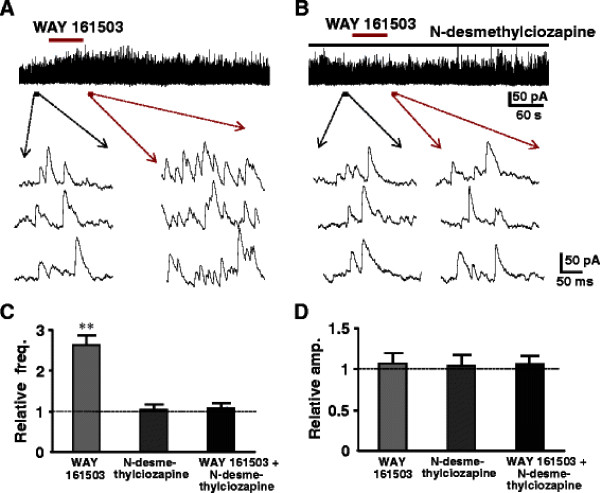
**Effects of 5-HT**_**2C**_**receptor agonist and antagonist on sIPSCs.** (**A**) Sample trace of sIPSCs recorded before, during and after the application of WAY 161503 (30 μM). Lower traces show sIPSCs before (left) and after (right) the application of WAY 161503 in the expanded time scale. (**B**) In the same (A) neuron, sample trace of sIPSCs recorded before (N-desmethylciozapine 10 μM), during and after the application of WAY 161503 in the presence of N-desmethylciozapine. Lower traces show sIPSCs before (left) and after (right) the application of N-desmethylciozapine. The bar graph shows the relative frequency (**C**) and amplitude (**D**) for WAY 161503, N-desmethylciozapine and WAY 161503 + N-desmethylciozapine, respectively (paired *t*-test, ** P < 0.01, n = 7).

**Figure 7:**
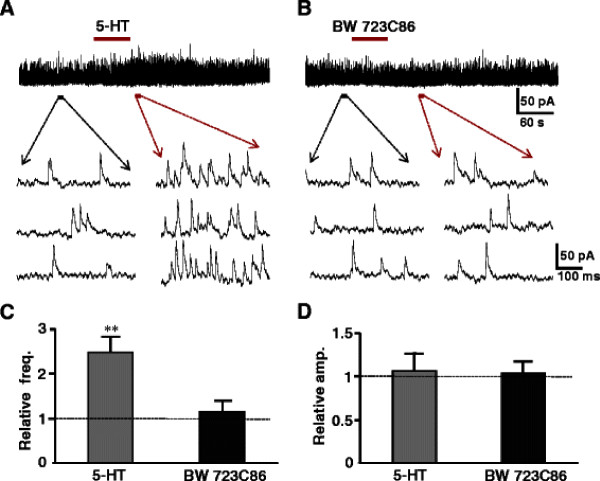
**Effects of 5-HT**_**2B**_** receptor agonist and antagonist on sIPSCs.** (**A**) Sample trace of sIPSCs recorded before, during and after the application of 5-HT (50 μM). Lower traces show sIPSCs before (left) and after (right) the application of 5-HT in the expanded time scale. (**B**) shows the same neuron (A) sample trace of sIPSCs recorded before, during and after the application of 5-HT_2B_ receptor agonist BW 723 C86 (10 μM). Lower races show sIPSCs before (left) and after (right) application of BW 723 C86. The bar graph shows the relative frequency (**C**), and amplitude (**D**) for 5-HT and BW 723 C86, respectively (paired *t*-test, ** P < 0.01, n = 7).

**Figure 8:**
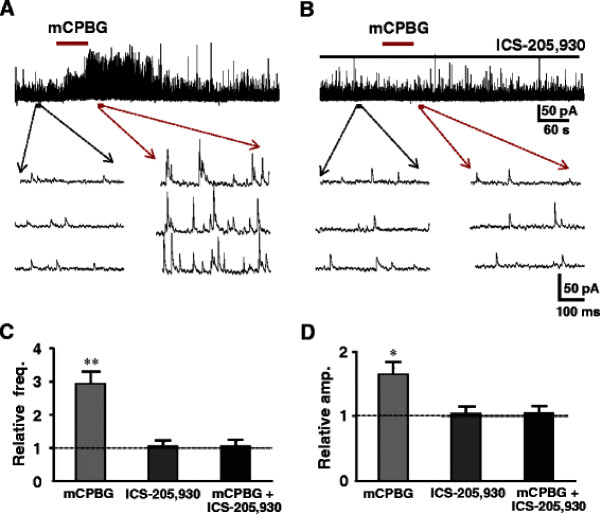
**Effects of 5-HT**_**3**_** receptor agonist and antagonist on sIPSCs.** (**A**) Sample trace of sIPSCs recorded before, during and after the application of mCPBG (30 μM). Lower traces show sIPSCs before (left) and after (right) the application of mCPBG in the expanded time scale. (**B**) In the same neuron from (A), sample trace of sIPSCs recorded before ICS-205,930 (10 μM), during and after the application of mCPBG in the presence of ICS-205,930. Lower traces show sIPSCs before (left) and after (right) the application of mCPBG in the presence of ICS-205,930 in the expanded time scale. The bar graph shows the relative frequency (**C**) and amplitude (**D**) for mCPBG, ICS-205,930 and mCPBG + ICS-205,930, respectively (paired *t*-test, ** P < 0.01 and *P < 0.05, n = 7).

Selective 5-HT receptor agonists and antagonists were tested to identify which subtypes of 5-HT receptors were responsible for the enhancement of the release of inhibitory neurotransmitters. 5-HT_1A_, 5-HT_2_, 5-HT_3_ and 5-HT_7_ subtypes are shown to expressed in the superficial dorsal horn neurons and terminals. 5-HT_1_ and 5-HT_7_ receptors are coupled to Gi/o, suggesting inhibitory effects. In fact, the 5-HT-induced membrane hyperpolarization or outward current in SG is mediated by 5-HT_1A_[13]. Based on these observations, we evaluated the effects of agonists and antagonists for 5-HT_2_, 5-HT_3_ and 5-HT_7_ receptor subtypes, in particular 5-HT_2A_, 5-HT_2C_ are expressed in the superficial spinal dorsal horn and behavioral studies show that intrathecal administration of 5-HT_2_ receptor agonist exhibit analgesic effects [48,49]. It is, however, still obscure which subtypes of 5-HT_2_ receptors are involved. We firstly tested each agonist for all subtypes of 5-HT_2_ receptors, and then corresponding antagonists were added to the perfusion to confirm the responsible subtypes with certainty. The agonists for 5-HT_2A_ receptor (TCB-2) and 5-HT_2B_ (BW 723C86) as well as 5-HT_2C_ receptors (WAY 161503) were given to the neurons whose sIPSCs were enhanced by prior application of 5-HT (Figures 5,6,7). The 5-HT_2A_ receptor agonist, TCB-2 (10 μM) mimicked the enhancing effects of 5-HT on sIPSCs, increasing the frequency without affecting the amplitude (Figure 5A). The frequency and amplitude of sIPSCs were, respectively, from 7.7 ± 4.0 Hz to 18.6 ± 5.6 Hz (n = 7, *P* < 0.01) and 19.6 ± 3.2 pA to 20.1 ± 3.4 pA (*P* > 0.05). The averages of the relative frequency and amplitude were 242% and 103% of control, respectively (Figure 5C, D). Perfusion of 5-HT_2A_ receptor selective antagonist, ketanserin (10 μM), itself had no detectable effect on the frequency and amplitude of sIPSCs (Figure 5B), markedly reduced the effects of the 5-HT_2A_ agonist. The averages of the relative frequency and amplitude were 104% and 102%, respectively, of their pre-application levels (Figures 5C, D). The 5-HT_2C_ receptor agonist, WAY 161503 (30 μM) also mimicked the 5-HT effect, enhanced the frequency but not amplitude of sIPSCs from 5.6 ± 1.0 Hz to 13.7 ± 1.9 Hz (*P* < 0.01) and 17.9 ± 0.8 pA to 19.1 ± 0.7 pA (Figure 6A, n = 7, *P* > 0.05), respectively. The averages of the relative frequency and amplitude were 245% and 106% of control, respectively (Figure 6C, D). In the presence of 5-HT_2C_ receptor selective antagonist, N-desmethylclozapine (10 μM), perfusion of WAY 161503 had no effect (Figure 6B). The frequency and amplitude were 107% (Figure 6C*P* > 0.05) and 104% of control (Figure 6D*P* > 0.05), respectively. Figure 7A showed that bath applied 5-HT increased the frequency but not amplitudes of sIPSCs from 4.6 ± 0.6 Hz to 11.4 ± 2.0 Hz (*P* < 0.01, 248% of control, n = 7), and 18.9 ± 1.3 pA to 20.2 ± 1.5 pA (*P* > 0.05, 106% of control), while an agonist of 5-HT_2B_ receptor, BW 723 C86 (10 μM) induced no change in both frequency (to 5.9 ± 0.8 Hz, 115% of control, *P* > 0.05, Figure 7C) and amplitude (to 19.8 ± 1.6 pA, 104% of control, n = 7, *P* > 0.05, Figure 7D) of sIPSCs recorded from the same neuron. The results demonstrated that 5-HT_2A_ and 5-HT_2C_ receptors might locate in the presynaptic terminals of inhibitory interneurons and responsible for the analgesic role of 5-HT. The 5-HT_3_ receptors are also showed to exist in the superficial dorsal horn and correlates with the analgesic role of 5-HT. Thus we further studied a role of 5-HT_3_ receptor agonist mCPBG (30 μM). The agonist mimicked the effects of 5-HT to increase both frequency and amplitude of sIPSCs from 6.8 ± 2.9 Hz and 22.9 ± 1.5 pA to 19.2 ± 7.6 Hz (292% of control, *p* < 0.01) and 37.3 ± 1.9 pA (165% of control, *p* < 0.05, n = 7), respectively (Figure 8A). Both effects were blocked completely by a 5-HT_3_ receptor antagonist ICS-205,930 (10 μM) (Figure 8B). The averages of the frequency and amplitude were 105% and 103% of their pre-application levels, respectively (Figure 8C, D). In the presence of TTX (0.5 μM), mCPBG enhanced the frequency but not amplitude (data not shown). The results suggested that 5-HT_3_ receptors were expressed at both on presynaptic terminals and soma-dendritic sites of inhibitory interneurons. In addition, we also tested the effect of 5-HT_1A_/5-HT_7_ receptors agonist (8-OH-DPAT 10 μM). The agonist induced no change in both frequency (to 5.7 ± 0.6 Hz, 107% of control, *P* > 0.05) and amplitude (to 20.2 ± 1.4 pA, 105% of control, n = 7, *P* > 0.05 data not shown).

## Discussion

In the present study, using blind whole cell recordings from adult rat spinal cord slices, the effects of 5-HT on inhibitory transmission were studied in SG to identify the receptor subtypes responsible for enhancement of release of GABA or glycine. The results showed that 5-HT modulated the sensory transmission in SG by the activation of 5-HT_2A_, 5-HT_2C_ and/or 5-HT_3_ receptors, possibly by reducing the potassium conductance as has been reported in the variety of CNS neurons.

Substantial numbers of 5-HT receptor subtypes predominate in the superficial laminae of the spinal dorsal horn, including 5-HT_1A_, 5-HT_2_, 5-HT_3_ and 5-HT_7_[19,49-53]. Among 5-HT receptor subtypes, 5-HT_1A_ and 5-HT_7_ receptors are coupled to Gi/o, suggesting an inhibitory effects [13,14,54,55]. In contrast 5-HT_2_ receptor is coupled to Gq/11, and 5-HT_3_ receptor is directly linked to nonselective cationic channels, suggesting an excitatory effects [13,47,49].

Consistent with this, an agonist for 5-HT_1A_ and 5-HT_7_, 8-OH-DPAT produced an outward current and did not show any significant effect on the inhibitory transmission (data not shown), indicating the 5-HT_1A_ and 5-HT_7_ receptors might not be responsible for the enhancement of GABA and glycine releases in the superficial dorsal horn. We showed that the activation of 5-HT_3_ receptors enhanced both frequency and amplitude, and TTX eliminated the effect on the amplitude, indicating that the 5-HT_3_ receptors were expressed at both terminals and soma-dendritic trees of inhibitory interneurons. It could not, however, exclude that 5-HT_2A_ and 5-HT_2C_ receptors were also expressed on both presynaptic terminals and soma dendrites; application of the agonists we used for 5-HT_2A_ and 5-HT_2C_ would not be high enough to initiate spike firing at somas, because of difference in density of receptors or efficacy of the agonists.

GABA and glycine are primary inhibitory transmitters in the spinal dorsal horn and play a critical role in modulating nociceptive transmission [37,40,56-58]. At the spinal level, plenty of reports demonstrate that the analgesic effects via 5-HT_2_ and 5-HT_3_ receptors are mediated by GABA [22,59-61], but a few reports concern with glycine. The present our study showed that not only GABAergic but also glycinergic transmission were augmented by 5-HT. The inconsistent results might be correlated with the location of the GABAergic and glycinergic neurons in the spinal dorsal horn. The previous studies show that glycinergic transmission are more prominent in laminae III-IV, whereas GABAergic transmission seem to predominate in lamina II [45,62]. In contrast to GABAergic neurons, the cell bodies of glycinergic neurons are preferentially located in lamina III and deeper laminae [40,42]. The behavioral studies show that intrathecal administration of 5-HT receptors agonists generally induce the inhibitory effects on the nociception, which can be blocked by the corresponding antagonists [27,29-31,33,63]. Accordingly, these results suggest that 5-HT_2_ and 5-HT_3_ receptors located in the spinal dorsal horn are involved in the antinociception. Pile of evidence shows that 5-HT exhibits both inhibitory and excitatory effects with complex mechanisms, and mechanisms are not fully understood up to now as there are too many subtypes of 5-HT receptor. The complexity in the effects of 5-HT might be concerned with 5-HT activating distinct subtypes of 5-HT receptor or affecting different types of neurons. Recent reports show that SG is composed at least of four types neurons, islet, small islet, vertical and radial neurons. The islet neurons are inhibitory interneurons and the small islet neurons include both excitatory and inhibitory interneurons [13,64,65]. It is reported that 5-HT depolarizes a small population of SG neurons which is morphologically classified into islet cell type [13].

The previous electrophysiological studies show that 5-HT exerts different postsynaptic effects on different types of SG neurons. Depolarization is induced in 6.8% neurons mimicked by 5-HT_3_ receptor agonist mCPBG and block by 5-HT_3_ receptor antagonist ICS-205,930 [13]. In DRG neurons, the activation of 5-HT receptors also induces a rapid depolarization. Also slice studies show that 5-HT potentiates the GABA or glycine-induced Cl^-^-current in the rat sacral dorsal commissural nucleus and superficial spinal dorsal horn [60,66-68].

In recent studies, behavioral nociceptive tests show that the 5-HT_7_ receptor play an antinociceptive role at the level of the spinal cord [26,54,55]. Immunocytochemical studies found that 5-HT_7_ receptors are localized in the superficial spinal dorsal horn [17,18]. However, our electrophysiological studies did not show that the 5-HT_7_ receptor modulated the inhibitory transmission in SG. This result was confirmed by perfusion of 8-OH-OPAT (10 μM), a mixed 5-HT_1A_/5-HT_7_ receptors agonist had no effect on sIPSCs (Data not shown).

## Conclusions

The present study demonstrated that 5-HT could enhance the release of GABA and glycine by activating the 5-HT_2A_, 5-HT_2C_ and/or 5-HT_3_ receptors expressed on inhibitory interneurons to inhibit sensory transmission. The 5-HT_2A_ and 5-HT_2C_ receptors predominantly exist at presynaptic terminals, while the 5-HT_3_ receptor might exist at both cell bodies and terminals. It is reported that 5-HT_2A_ receptors also located on soma-dendritic trees [69]. But our result did not coincide with the report. The contradiction might be concerned with the distribution density of the receptors.

In short, the present study provided more evidences to explain the various mechanisms of 5-HT on modulating nociceptive transmission in SG of the spinal dorsal horn.

## Methods

All the experimental procedures involving the use of animals were approved by the Ethics Committee on Animal Experiments, Kyushu University, and were in accordance with the UK Animals (Scientific Procedures) Act 1986 and associated guidelines. All efforts were made to minimize the number of animals used for the studies.

### Spinal cord slice preparation

Methods for obtaining adult rat spinal cord slices and for bind patch-clamp recordings from SG neurons were identical to those described elsewhere [70,71]2. Briefly, male adult Sprague–Dawley rats (6–7 weeks) were deeply anesthetized with urethane (1.2g/Kg, ip), and then thoracolumbar laminectomy was performed. The lumbosacral spinal cord was removed and placed in a preoxygenated cold Krebs solution containing (in mM): NaCl 117, KCl 3.6, CaCl_2_ 2.5, MgCl_2_ 1.2, NaH_2_PO_4_ 1.2, NaHCO_3_ 25 and glucose 11 at 1-3°C. The pia-arachnoid membrane was removed after cutting all the ventral and dorsal roots. The spinal cord was mounted on a vibratome and then a 500 μm thick transverse or parasagittal slice was cut. The slice was placed on a nylon mesh in the recording chamber and then perfused at a rate of 15–20 ml/min with Krebs solution saturated with 95% O_2_ and 5% CO_2_, at 36 ± 1°C.

### Patch-clamp recordings from substantia gelatinosa neurons

Blind whole-cell voltage-clamp recordings were made from SG neurons with patch pipettes filled with a solution containing (mM): Cs_2_SO_4_ 110, tetraethylammonium (TEA) 5, CaCl_2_ 0.5, MgCl_2_ 2, EGTA 5, HEPES 5 and ATP-Mg 5 (PH 7.2). Cs_2_SO_4_ and TEA were main chemicals which could inhibit the postsynaptic effects of 5-HT on the K^+^ channels, and enabled us to investigate the presynaptic effects on IPSCs by 5-HT. Recorded neurons were identified as SG by their locations and morphologic features. SG was easily identifiably as a relatively translucent band across the dorsal horn. In some instances, neurobiotin was injected in the recorded neurons through electrodes. After completing experiments, the recorded neuron were stained and their morphological features were compared with those reported previously [13,64,65,72]. Monosynaptic IPSCs were evoked in the presence of a non-NMDA-receptor antagonist CNQX, and an NMDA-receptor antagonist APV, at a frequency of 0.2 Hz by a focal monopolar silver electrode (50 μm diameter), insulated except for the tip, located within 150 μm of the recorded neurons. Signals were acquired with a patch clamp amplifier (Axopatch 200B, Molecular Devices, Union City, CA, USA). The data were digitized with an analog-to-digital converter (digidata 1321A, Molecular Devices, CA, USA), and stored and analyzed with a personal computer using the pCLAMP data acquisition program (version 8.2, Molecular Devices, CA, USA). The recordings were made under the voltage-clamp mode at holding membrane potentials of 0 mV to isolate IPSCs. At this potential the glutamate-mediated excitatory postsynaptic currents (EPSCs) were negligible, because of a reversal potential of EPSCs. In fact, no remaining synaptic currents were observed in the presence of antagonists for GABA and glycine receptors [70]. Frequencies and amplitudes of spontaneous IPSCs (sIPSCs) and miniature IPSCs (mIPSCs) in the presence of TTX (0.5 μM) were measured automatically with MiniAnalysis software (Synaptosoft, Decatur, GA). The frequency of IPSCs was further confirmed by their shapes with eyes.

### Drug application

Drugs dissolved in Krebs solution were applied to the surface of the spinal cord by exchanging solutions via a three-way stopcock without any change in both perfusion rate and temperature. The time necessary for the solution to flow from the stopcock to the surface of the spinal cord was approximately 5 s and the solution in the recording chamber was completely exchanged with a drug containing solution within 15 s. The drugs used were 5-HT hydrogen maleate (Sigma, St. Louis, MO, USA), tetrodotoxin (TTX) (Wako, Osaka, Japan), 6-cyano-7-nitroquinoxaline-2,3-dione (CNQX) (Sigma), DL-2-amino-5-phosphonovaleric acid (APV) (Sigma), BaCl_2_ (Sigma), strychnine (Sigma), bicuculline (Sigma), 4-bromo-3,6-dimethoxybenzocyclobuten-1-yl) methylamine hydrobromide (TBC-2) (Tocris Cookson, Bristol, UK), ketanserin (Sigma), α-methyl-5-(2-thienylmethoxy)-H-indole-3-ethanamine hydrochloride (BW723C86) (Sigma), 8,9-dchloro-2,3,4 4a-tetrahydro-1H-pyrazino[1,2-a] quinoxalin-5(6H)-one hydrochloride (WAY161503) (Tocris), 8-chloro-11-(1-piperazinyl)-5H-dibenzo[b,e][1,4]diazipine (N-desmethylclozpine) (Tocris), 1-(m-chlorophenyl)-biguanide (mCPBG) (Sigma), 3-tropanylindole-3-carboxylate methiodide (ICS-205,930) (Sigma), (±)-8-hydroxy-2-dipropylaminotetralin hydrobromide (8-OH-DPAT) (Sigma).

### Statistical analysis

All the data were expressed as the mean ± S.E.M. Statistical significance was determined as *P* < 0.05 using the paired *t*-test. Cumulative probability plots were constructed for sIPSC amplitude and frequency and were compared, under different experimental conditions, using the Kolmogorov-Smirnov test. In all cases, n refers to the number of neurons studied.

## Abbreviations

5-HT, 5-hydroxytryptamine; CNS, central nervous system; SG, substantia gelatinosa; sIPSCs, spontaneous inhibitory postsynaptic currents; eIPSCs, evoked IPSCs; RVM, rostral ventromedial medulla; GABA, γ-aminobutyric acid; TEA, tetraethylammonium; EPSCs, excitatory postsynaptic currents; TTX, tetrodotoxin; CNQX, 6-cyano-7-nitroquinoxaline-2,3-dione; APV, DL-2-amino-5-phosphonovaleric acid; TBC-2, 4-bromo-3,6-dimethoxybenzocyclobuten-1-yl) methylamine hydrobromide; BW723C86, α-methyl-5-(2-thienylmethoxy)-H-indole-3-ethanamine hydrochloride; WAY161503, 8,9-dchloro-2,3,4 4a-tetrahydro-1H-pyrazino[1,2-a] quinoxalin-5(6H)-one hydrochloride; N-desmethylclozpine, 8-chloro-11-(1-piperazinyl)-5H-dibenzo[b,e][1,4]diazipine; mCPBG, 1-(m-chlorophenyl)-biguanide; ICS-205,930, 3-tropanylindole-3-carboxylate methiodide; 8-OH-DPAT, (±)-8-hydroxy-2-dipropylaminotetralin hydrobromide.

## Competing interests

The authors declare that we have no competing interests.

## Authors’ contributions

DJX carried out all of the experiments and majority data analysis. DU, MW, MCS participate in some of the data analysis. PYF, HF, MY and DJX conceptualized the project and formulated the hypothesis and wrote the manuscript. MY designed and directed the experiments. All authors read and approved the final manuscript.
